# Pretreatment prognostic nutritional index as a prognostic marker in head and neck cancer: a systematic review and meta-analysis

**DOI:** 10.1038/s41598-021-96598-9

**Published:** 2021-08-24

**Authors:** Chih-Wei Luan, Yao-Te Tsai, Hsin-Yi Yang, Kuan-Yin Chen, Po-Hsien Chen, Hsin-Hsu Chou

**Affiliations:** 1 Department of Otorhinolaryngology-Head and Neck Surgery, LO-Sheng Hospital Ministry of Health and Welfare-Home, New Taipei City, Taiwan; 2 Clinical Medicine Research Center, Ditmanson Medical Foundation Chia-Yi Christian Hospital, Chiayi, Taiwan; 3 Graduate Institute of Clinical Medical Sciences, College of Medicine, Chang Gung University, Taoyuan, Taiwan; 4 Department of Otorhinolaryngology-Head and Neck Surgery, Chang Gung Memorial Hospital, Chiayi, Taiwan; 5 School of Dentistry, National Yang Ming University, Taipei, Taiwan; 6 Department of Pediatrics, Ditmanson Medical Foundation Chia-Yi Christian Hospital, 539, Zhongxiao Road, 600, Chiayi, Taiwan; 7 Department of Bioinformatics and Medical Engineering, Asia University, Taichung, Taiwan

**Keywords:** Cancer, Cell biology, Molecular biology, Oncology, Risk factors

## Abstract

The predictive value of the pretreatment prognostic nutritional index (PNI) for head and neck cancer (HNC) remains controversial. We conducted a meta-analysis to assess the predictive value of PNI in HNC patients. A systematic search through internet databases including PubMed, Embase, and Cochrane Library for qualified studies estimating the association of PNI with HNC patient survival was performed. Overall survival (OS), progression-free survival (PFS), disease-specific survival (DSS), disease-free survival (DFS) and distant metastasis-free survival (DMFS) data were collected and evaluated. A random-effects model was used to calculate the pooled hazard ratios (pHRs) and corresponding 95% confidence intervals (CIs). A total of 7815 HNC patients from 14 eligible studies were involved. Pooled analysis showed that low pretreatment PNI was correlated with poor OS (pHR: 1.93, 95% CI 1.62–2.30, *p* < 0.001), PFS (pHR: 1.51, 95% CI 1.19–1.92, *p* = 0.008), DSS (pHR: 1.98, 95% CI 1.12–3.50, *p* < 0.001), DFS (pHR: 2.20, 95% CI 1.66–2.91, *p* < 0.001) and DMFS (pHR: 2.04, 95% CI 1.74–2.38, *p* < 0.001). Furthermore, low pretreatment PNI was correlated with poor OS despite variations in the cancer site, sample size, PNI cut-off value, analysis method (multivariate analysis or univariate analysis) and treatment modality in subgroup analysis. Elevated pretreatment PNI is correlated with a superior prognosis in HNC patients and could be used as a biomarker in clinical practice for prognosis prediction and treatment stratification.

## Introduction

Head and neck cancer (HNC), which includes several malignancies arising from the oral cavity, nasopharynx, oropharynx, hypopharynx and larynx, is an important issue of public health worldwide. More than 400,000 people died of HNC in 2018, and 800,000 cases were newly diagnosed each year^1^. Multidisciplinary approach to patients with HNC at different stage and situation was based on institution guidelines, while two main strategies were widely performed. Surgical resection combined with or without adjuvant therapy based on clinical and pathological risk factors and radiotherapy with or without chemotherapy remain the standard treatment for HNC. Despite advances in multidisciplinary management and aggressive treatment in HNC, the prognosis is still unsatisfactory, and the survival outcomes of patients with the same tumour-node-metastasis (TNM) stage usually vary^2^. Therefore, understanding tumour carcinogenesis and identifying available serum biomarkers of HNC could contribute to personalized treatment with better prognosis prediction and patient stratification^3,4^.

Increased evidence has shown that host immunity and systemic inflammation play an important role in cancer angiogenesis and progression, although the exact pathogenesis is not thoroughly understood^5,6^. Accumulated studies have shown that many inflammation-based scores, including the neutrophil-to-lymphocyte ratio, Glasgow prognostic score, lymphocyte-to-monocyte ratio, platelet-to-lymphocyte ratio, and C-reactive protein to albumin ratio, are associated with cancer survival and prognosis^7,8^. Identification of precise and available prognostic indicators may optimize the management of patients suffering from HNC. The prognostic nutritional index (PNI) incorporating serum albumin level and peripheral blood lymphocyte count serves as an indicator of systemic inflammation and host nutritional status. Several studies conducted PNI as an independent prognostic biomarker in cancers, including oesophageal squamous cell carcinoma, small or non-small-cell lung cancer, pancreatic cancer, gastric cancer and colorectal cancer^9–15^. A meta-analysis investigated the relationship between PNI and tumour survival through 14 published studies and found that a low PNI level was associated with poor overall survival (OS) in terms of tumour type, surgical condition, cut-off value, sample size and area^16^. However, no HNC patients were included in this meta-analysis. Therefore, conducting a comprehensive meta-analysis may provide evidence supporting clinical practice and the formation of clear clinical guidelines for HNC. While the assessment of some haematological biomarkers involves expensive testing, the PNI is a readily available and cost-effective marker in the pretreatment evaluation of patients via blood tests. Thus, we performed this meta-analysis by pooling the related literature to quantify the prognostic value of PNI on survival outcomes in patients with HNC.

## Results

### Study characteristics

**Figure 1:**
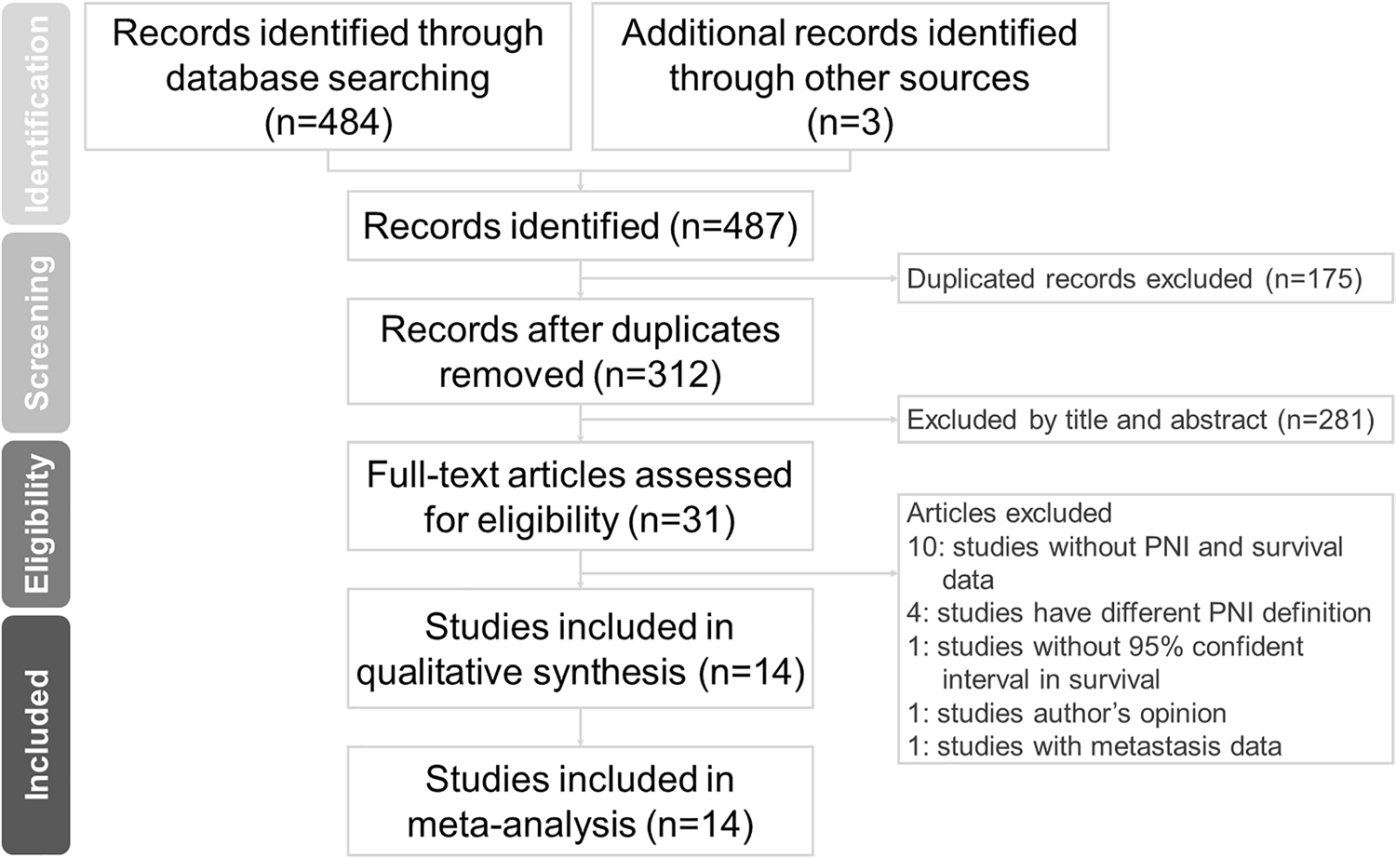
Flow diagram of study selection.

A total of 484 records were identified through a literature search of the PubMed, Embase and CENTRAL databases. Figure 1 shows the process of the literature search, and 175 duplicate studies were excluded. Following the titles and abstracts screened, 31 full-text articles lasted for further appraisal, and 17 articles were excluded based on our inclusion and exclusion criteria. Finally, 14 articles comprising 7815 patients qualified for accessible synthesis^17–30^.

Table 1 displays the demographics and characteristics of the included studies. The sample sizes of these studies ranged from 95 to 1395, and all included studies were published between 2015 and 2020. The cut-off values of PNI varied from 40 to 56.93; those for 3 studies were derived by using Cut-off Finder, and those for 2 studies were set as the mean value. A receiver operating characteristic curve was used in 8 studies. The last study used the previously reported value of 40 as the cut-off value. All these included studies with NOS scores ≥ 7 were considered to be of good quality. (Supplemental Table 1).

**Table 1:** Demographic characteristics of the included studies.

First author	Year	Country	Sample size	Tumor site	Cancer stage (AJCC)	Curative/palliative	Treatment	Cut-off value resource	PNI cut-off	Outcome	Median follow-up (months)	NOS
He et al	2019	China	377	NPX	II–IV*	Curative	CCRT	Cutoff Finder	49.05	OS/PFS	40	7
Yang et al	2016	China	1168	NPX	I–IV*	Curative	RT/CCRT	Cutoff Finder	51	DMFS	68.8	7
			756	NPX	I–IV*	Curative	RT/CCRT	Cutoff Finder	51	DMFS	60.25	
Gundog et al	2019	Turkey	95	NPX	II–IVa	Curative	CCRT	ROC curve	45.45	OS/DMFS	41	7
Oei et al	2018	China	585	NPX	I–IV*	Curative	RT/CCRT	ROC curve	53	OS/PFS/DMFS	63.3	8
Miao et al	2017	China	220	NPX	II–IVB*	Curative	RT/CCRT	ROC curve	52	OS/DSS/DMFS	109.5	8
			270	NPX	II–IVB*	Curative	RT/CCRT	ROC curve	52	OS/DSS/DMFS	109.5	
Du et al	2015	China	694	NPX	I–III	Curative	RT/CCRT	Median value	55	OS/PFS/DMFS	88	7
Zeng et al	2020	China	559	NPX	I–IV*	Curative	RT/CCRT	ROC curve	45.58	OS/DFS	33.5	7
Ikeguchi et al	2016	Japan	59	HPX	III–IV*	Curative	OP + CCRT	Pre-study	40	OS	28	7
Ye et al	2018	China	123	HPX	I–IV*	Curative	OP/OP + CCRT	Median value	52	OS/PFS/DFS/DMFS	39.5	7
Fu et al	2016	China	975	LAX	I–IV*	Curative	OP/OP + CCRT	Cutoff Finder	56.93	OS	83	8
Fu et al	2019	China	61	LAX	III–IV*	Curative	RT/CCRT	ROC curve	44	OS	NR	8
Bao et al	2020	China	1395	ORAL	I–IV*	Curative	Mixed	Median value	49.3	OS/DSS	NR	7
Wu et al	2020	China	166	ORAL	I–IV*	Curative	Mixed	ROC curve	47.4	OS/DFS	NR	7
			167	ORAL	I–IV*	Curative	Mixed	ROC curve	47.4	OS/DFS		
Bruixola et al	2018	Spain	50	MIX	III–IV*	Curative	CCRT	ROC curve	45	OS	NR	7
			95	MIX	III–IV*	Curative	CCRT	ROC curve	45	OS		

CT, chemotherapy; RT, radiotherapy; CCRT, concurrent chemo-radiotherapy AJCC = American Joint Committee on Cancer, PNI = prognostic nutrient index, PFS = progression free survival, DSS = disease specific survival, DFS = disease free survival, DMFS = distant metastasis-free survival, NOS = Newcastle–Ottawa Scale scores, NR = not reported, OS = overall survival, OP = operation, ROC = receiver operating characteristic curve, NPx = nasopharynx, HPx = hypopharynx, LAX = larynx, MIX = Mixed (all head and neck cancer).

*Exclude metastasis patient.

#### Prognostic value of OS in patients with HNC

**Figure 2:**
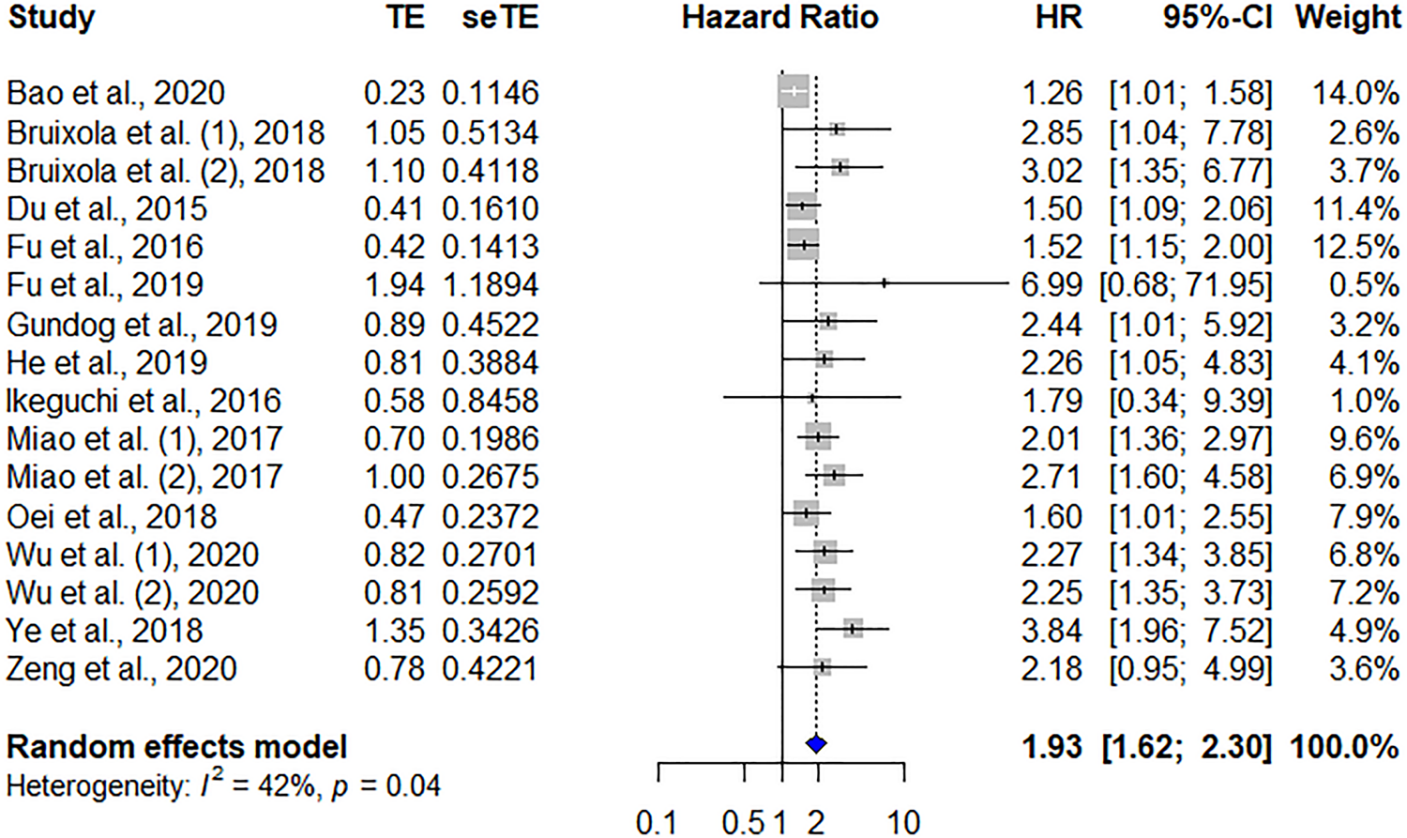
Forest plot showing the hazard ratios of PNI for predicting overall survival in patients with head and neck cancer. HR, hazard ratio; CI, confidence interval; PNI, prognostic nutritional index.

Fourteen studies comprising 5891 patients showed the predictive role of the pretreatment PNI for OS. The pooled HR was 1.93 (95% CI 1.62–2.30, *p* < 0.001) and showed that a low PNI was significantly correlated with poor OS in patients with HNC. Low heterogeneity was recognized between studies (*I*^2^ = 42%, *P*_heterogeneity_ = 0.04; Fig. 2).

#### Prognostic value of PFS in patients with HNC

**Figure 3:**
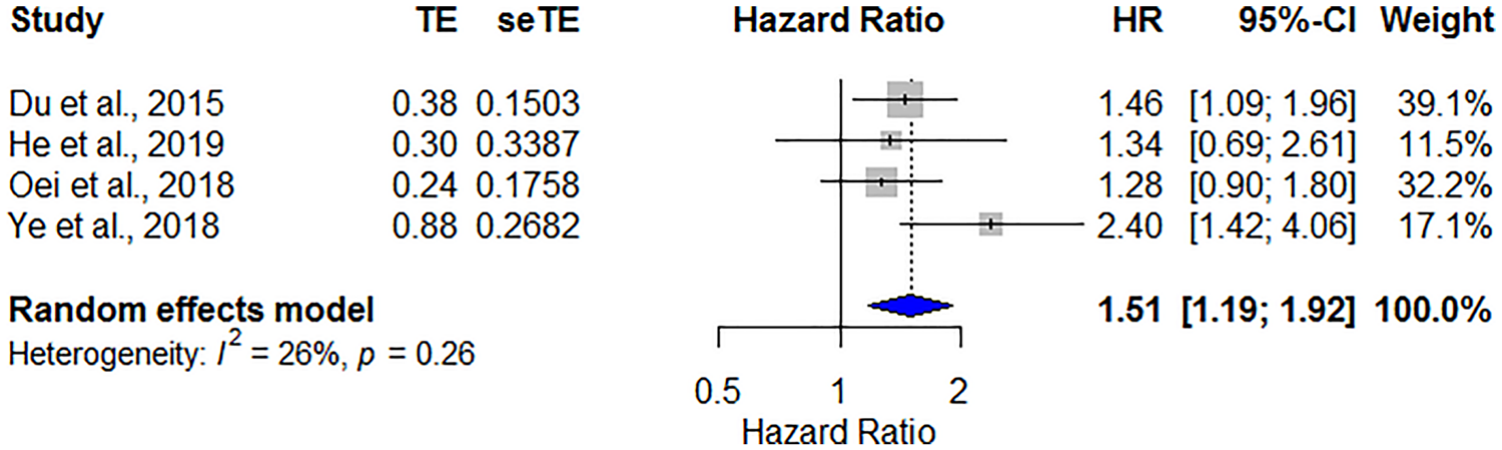
Forest plot showing the hazard ratios of PNI for predicting progression-free survival in patients with head and neck cancer. HR, hazard ratio; CI, confidence interval; PNI, prognostic nutritional index.

In total, four studies that enrolled 1779 patients assessed the prognostic effect of low pretreatment PNI on HNC. The pooled data revealed that a low PNI was significantly correlated with poor PFS (HR = 1.51, 95% CI 1.19–1.92, *p* = 0.008), and low heterogeneity was recognized between studies (*I*^2^ = 26%, *P*_heterogeneity_ = 0.26; Fig. 3).

#### Prognostic value of DFS in patients with HNC

**Figure 4:**
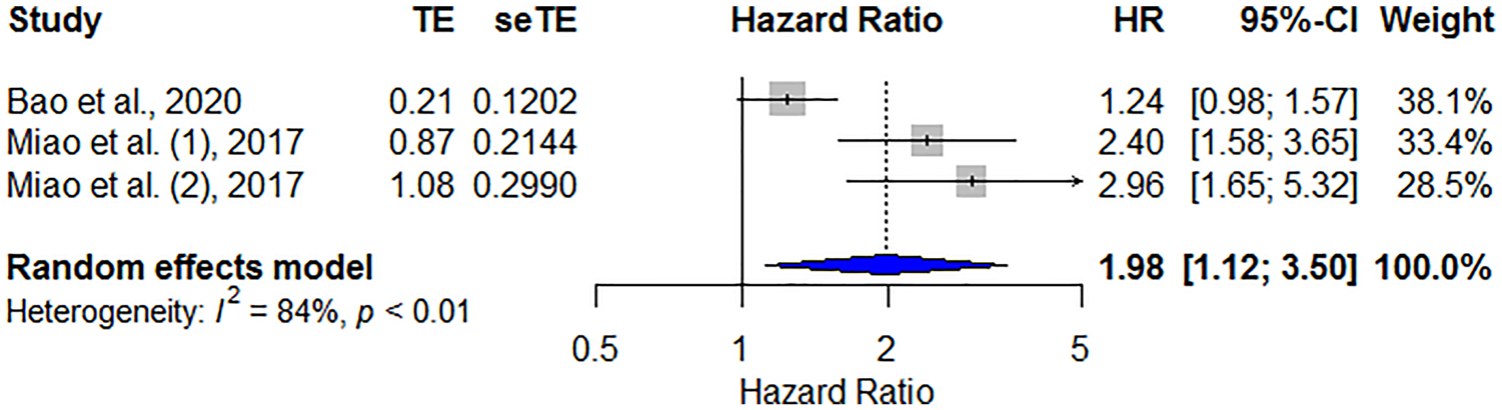
Forest plot showing the hazard ratios of PNI for predicting disease-free survival in patients with head and neck cancer. HR, hazard ratio; CI, confidence interval; PNI, prognostic nutritional index.

In general, three studies including 1015 patients presented HRs for DFS of PNI in patients with HNC. The pooled data indicated that a low PNI was significantly correlated with poor DFS (HR = 2.20, 95% CI 1.66–2.91, *p* < 0.001), and no heterogeneity was regarded between studies (*I*^2^ = 0%, *P*_heterogeneity_ = 0.76; Fig. 4).

#### Prognostic value of DSS in patients with HNC

**Figure 5:**
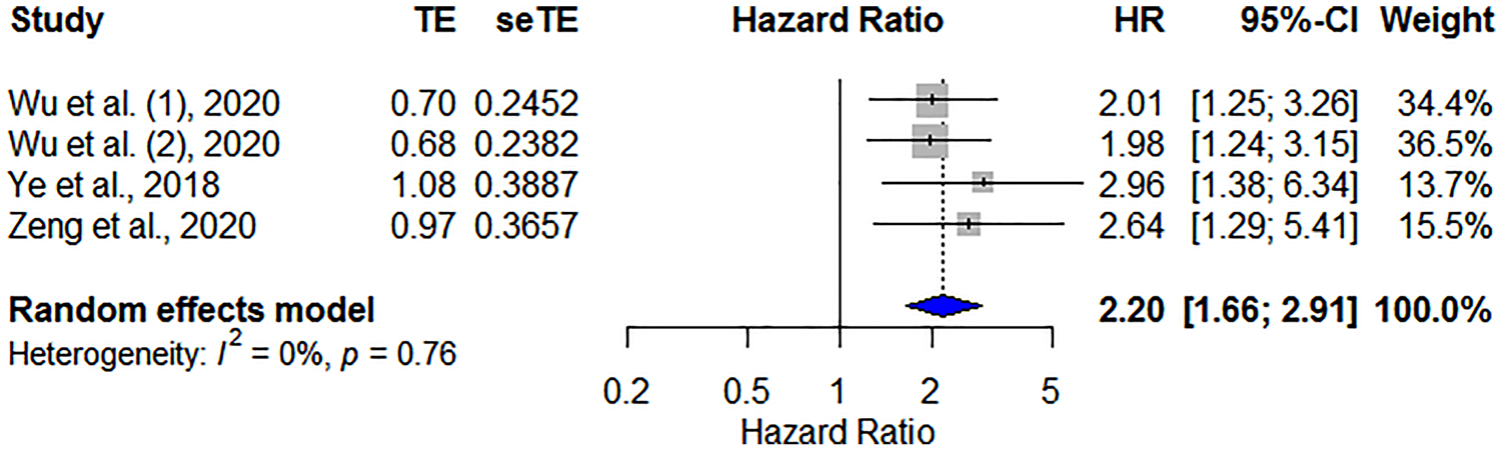
Forest plot showing the hazard ratios of PNI for predicting disease-specific survival in patients with head and neck cancer. HR, hazard ratio; CI, confidence interval; PNI, prognostic nutritional index.

Two studies included three groups of 1885 nasopharyngeal carcinoma (NPC) patients with HRs for DSS according to the PNI. The pooled data revealed that a low PNI was significantly correlated with poor DSS (HR = 1.98, 95% CI 1.12–3.50, *p* < 0.001), and high heterogeneity was observed between studies (*I*^2^ = 84%, *P*_heterogeneity_ < 0.01; Fig. 5).

#### Prognostic value of DMFS in patients with HNC

**Figure 6:**
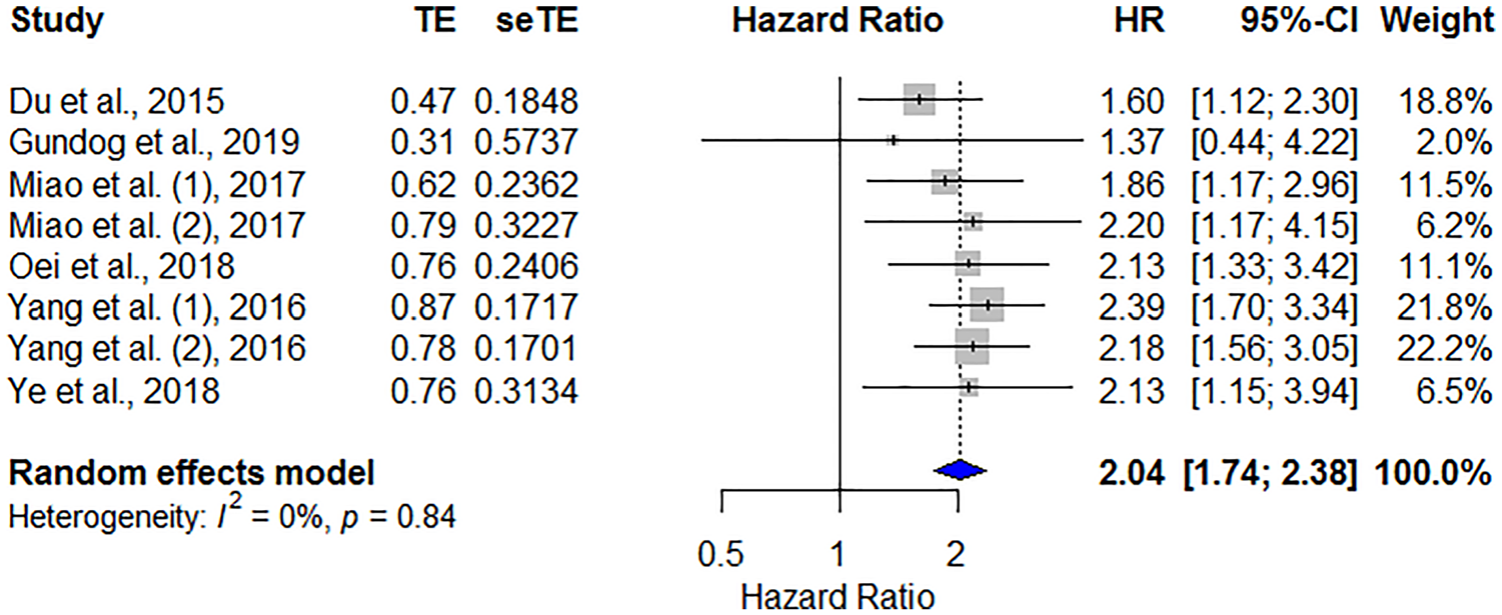
Forest plot showing the hazard ratios of PNI for predicting distant metastasis–free survival in patients with head and neck cancer. HR, hazard ratio; CI, confidence interval; PNI, prognostic nutritional index.

Overall, six studies including 3911 patients contributed to the HR for DMFS of PNI in patients with HNC. The pooled data showed that a low pretreatment PNI was significantly correlated with poor DMFS (HR = 2.04, 95% CI 1.74–2.38, *p* < 0.001), and no heterogeneity was observed between studies (*I*^2^ = 0%, *P*_heterogeneity_ = 0.84; Fig. 6).

#### Subgroup analysis

**Figure 7:**
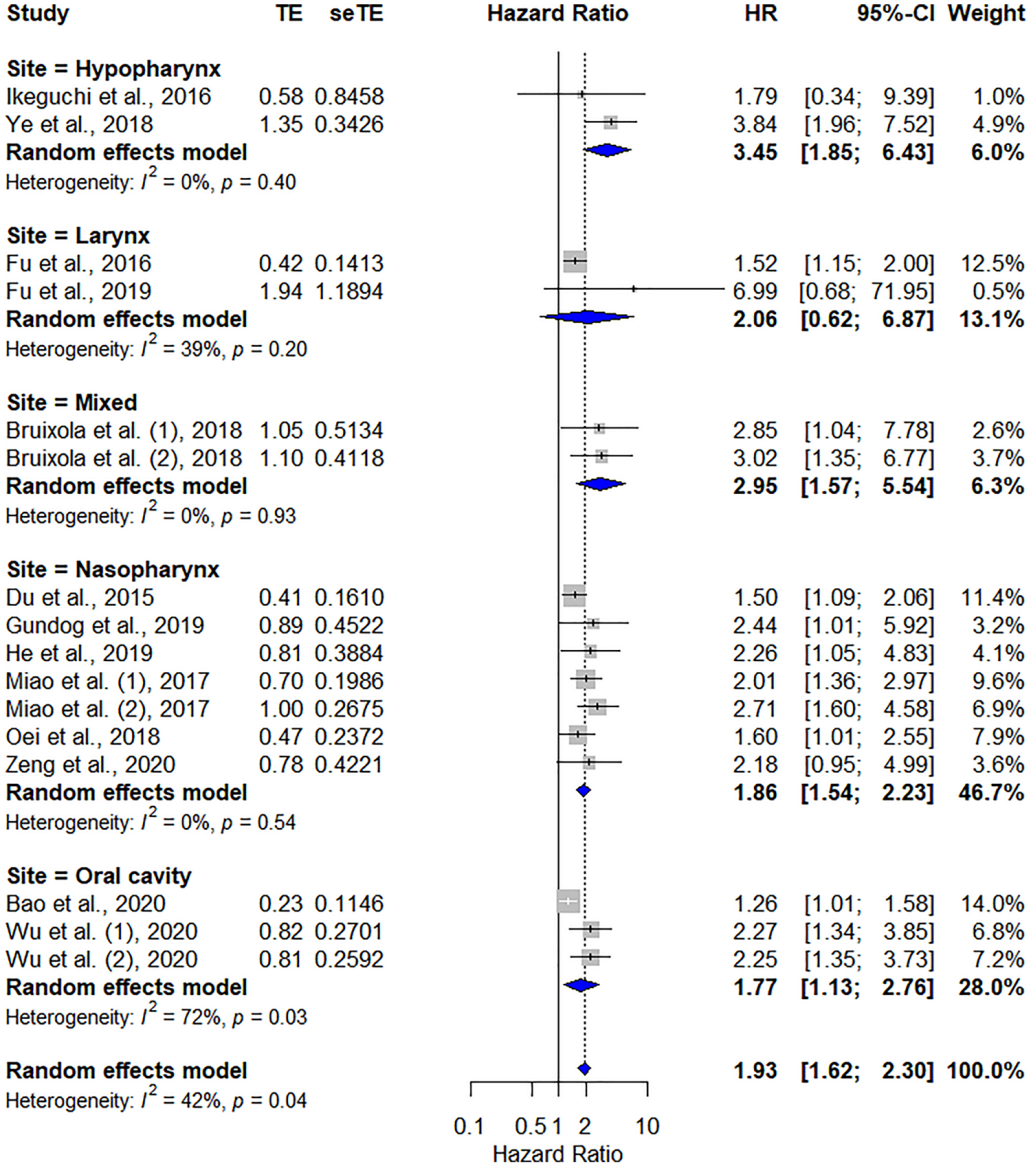
Forest plot depicting the pooled results of HRs for OS related to various tumour locations. HR, hazard ratio; CI, confidence interval; OS, overall survival.

Subgroup analyses were conducted to reduce the potential confounding factors. In the present study, heterogeneity in the OS analysis may have resulted from the inclusion of all subsites of HNC. Because HNC is a heterogeneous disease entity with tumours originating from the nasopharynx, oropharynx, hypopharynx, larynx and oral cavity, tumour location may influence the prognosis and survival of HNC. In the subgroup analysis concerning tumour site, the PNI revealed a significant correlation with OS and in all subsites except in those with laryngeal cancer (Fig. 7). In the individual subgroup analysis by tumour site, the heterogeneity among studies was generally low (nasopharynx, *I*^2^ = 0%; *P*_heterogeneity_ = 0.54; hypopharynx, *I*^2^ = 0%; *P*_heterogeneity_ = 0.4; larynx, *I*^2^ = 39%; *P*_heterogeneity_ = 0.2; mixed head and neck, *I*^2^ = 0%; *P*_heterogeneity_ = 0.93), except in the oral cavity (*I*^2^ = 72%; *P*_heterogeneity_ = 0.03), implicating that the inclusion of all primary tumour sites, especially the oral cavity, might be the leading reason for heterogeneity in the present study (Table 2).

**Table 2:** Subgroup analysis of the correlation of PNI and OS according to possible confounding factors.

Subgroup	Number of studies	Number of patients	Pooled HR with 95% CI	*p* value	Heterogeneity	
					*I*^2^ (%)	*p* value
Total	13	5891	1.93 (1.62–2.30)	< 0.001	41.6	0.041
**Tumor location**						
Nasopharynx	6	4345	1.85 (1.54–2.33)	< 0.001	0.0	0.536
Oral cavity	2	1728	1.77 (1.13–2.76)	0.013	71.6	0.030
Hypopharynx	2	182	3.45 (1.85–6.43)	< 0.001	0.0	0.402
Larynx	2	1036	2.06 (0.62–6.87)	0.238	38.7	0.202
Head and neck	1	145	2.95 (1.57–5.54)	< 0.001	0	0.928
**Treatment**						
OP + adjuvants	3	1157	2.18 (1.06–4.48)	0.035	68.3	0.043
RT or CCRT	5	3754	1.85 (1.49–2.29)	< 0.001	11.6	0.341
CCRT	3	1044	2.60 (1.70–3.97)	< 0.001	0.0	0.957
Others	2	1728	1.77 (1.13–2.76)	0.013	71.6	0.030
**Sample size**						
≤ 500	8	1683	2.44 (2.00–2.96)	< 0.001	0	0.931
> 500	5	4208	1.43 (1.24–1.65)	< 0.001	0	0.611
**Cut-off value for PNI**						
≤ 50	8	3024	2.05 (1.55–2.72)	< 0.001	39.6	0.094
> 50	5	2867	1.89 (1.48–2.41)	< 0.001	52.4	0.062
**Analysis method**						
Multivariate analysis	11	5273	1.94 (1.61–2.34)	< 0.001	48.7	0.021
Univariate analysis	2	618	2.10 (1.00–4.39)	0.050	0.0	0.834

OS, overall survival; PNI, Prognostic nutrition index; N status = lymphatic stage; OP, operation; RT, radiotherapy; CCRT, concurrent chemo-radiotherapy.

We also performed subgroup analyses to assess the impact of treatment modality, study sample size, PNI cut-off value, analysis method and disease stage on OS (Table 2). Low pretreatment PNI was correlated with poor OS in the concurrent chemoradiotherapy (CCRT) group and radiotherapy (RT) or chemotherapy group with no heterogeneity (*I*^2^ = 0%, *P*_heterogeneity_ = 0.96; *I*^2^ = 12%, *P*_heterogeneity_ = 0.34). High heterogeneity was found in the adjuvant therapy group (*I*^2^ = 68%, *P*_heterogeneity_ = 0.04) and the other therapy groups (*I*^2^ = 71.6%, *P*_heterogeneity_ = 0.03). Further analyses according to sample size, PNI cut-off value and analysis method were conducted, which revealed consistent pooled HRs in all subgroups and found that low pretreatment PNI was correlated with poor OS despite potential confounding factors. Of note, the effect size of small studies (sample size ≤ 500; HR 2.44, 95% CI 2.00–2.96) was greater than that of large studies (sample size > 500; HR 1.89, 95% CI 1.48–2.41).

#### Publication bias and sensitivity analysis

Publication bias was assessed using the funnel plot and Egger's test for OS (Figure S1). For the 14 studies, the funnel plot was visually asymmetric, and significant publication bias was found. (Egger’s test: *p* < 0.01). We employed the trim and fill method to evaluate the stability of the pooled results^31^. The pooled HR of OS (1.62, 95% CI 1.35–1.90, *p* =  < 0.001) changed little and remained the same direction after performing the trim and fill method. We conducted sensitivity analysis to evaluate the stability of the PNI in assessing OS by removing each study sequentially from the pooled analysis, and the pooled HR did not change significantly after the omission of any of the included studies (Supplemental Table 2). Our initial qualitative interpretation of the pooled result did not change by substitution of the random-effects model with the fixed-effect model.

## Discussion

Based on a review of the literature, our meta-analysis is the first study to elucidate the prognostic value of pretreatment PNI in patients with HNC. Our results were obtained by analysing data on 7815 HNC patients in 14 individual studies, and the pooled results revealed that a low pretreatment PNI was correlated with poor survival (OS, PFS, DFS, DSS, and DMFS) for patients with HNC. Among the included studies, low heterogeneity was observed in OS and PFS, and no significant heterogeneity was observed in DFS and DMFS. Notably, HNC includes squamous cell carcinoma of the nasopharynx, oropharynx, hypopharynx, larynx and oral cavity. Different tumour locations are an important factor that determines the treatment and prognosis of HNC. In the present study, we performed subgroup analysis with data from 14 studies with reported OS by tumour location, which significantly reduced the heterogeneity, implicating that the primary tumour site may be the main reason for heterogeneity and supporting our results regarding the impact of the PNI on OS.

The pathophysiological mechanisms of the correlation between PNI and cancer survival are still poorly understood. Bruixola et al. performed a retrospective cohort study and revealed that the PNI was significantly associated with primary tumour location, human papillomavirus (HPV) status, alcohol addiction and smoking and served as an independent prognostic marker in patients with HNC^21^. PNI combines both nutrition and systemic inflammation status, while a low PNI implies a decrease in lymphocytes and/or albumin, serving as surrogate biomarker. Albumin is an important nutritional status indicator; low serum albumin has been used as an independent indicator of poor survival in various cancer^32,33^. The peripheral blood lymphocyte count has been assumed to be an important contributor in preventing cancer by activating host immune response^34^. The inflammatory reaction against cancer results in the release of small inflammatory proteins, chemokines, cytokines, acute-phase proteins, and immune cells, which may impact on cancer cell growth, death and apoptosis, enhancing cell invasion, facilitating angiogenesis and metastasis^35–37^. Current evidence demonstrates that lymphocytopenia and malnutrition together may as act as predictor of a chronically immune system damage.

Despites advancement in therapy, the personalized regimen for each patient is still a challenging and often debatable choice. The treatment failure rate is still high due to locoregional recurrence and distant metastasis^38^. In general, poor nutritional and immune status usually increase treatment failure in cancer patients. Adjuvant therapy like RT or CCRT supposed to have a negative influence on nutritional status^39^. Malnutrition could damage immune system and increases patient susceptibility to infection^40^. Poor nutrition status or infection consequently causes patients to abandon RT/CCRT, which largely prevents cancer patients from benefiting from cancer management. Prognostic factors could advise physicians in choosing the most appropriate treatment for individual patient and inform patients about the long-term treatment outcomes. Taken together, the data collected in our study indicate that a low PNI is a risk indicator for a poor survival in HNC. In this respect, simple and practical biomarker may be valuable for identification and screening of HNC patients who could need a personalized diagnostic methods and therapeutic intervention.

Treatment strategy has also been shown to be a crucial prognostic factor in HNC. We performed subgroup analyses stratified by difference in treatment modality, a lower pretreatment PNI was related to a poorer OS according to the pooled HR (*p* < 0.05). There was high heterogeneity in the group with surgery following adjuvant therapy and the group with mixed therapy (based on the patient’s clinical stage and hospital treatment guidelines). Currently, comprehensive treatment is standard in HNC, and this approach was utilized in most of the included studies. Therefore, high heterogeneity across treatment strategies was observed in our study. Further subgroup analysis according to sample size was conducted to confirm the consistency of the findings, reduce sample size bias and further explore the connection between PNI and treatment strategy. When divided by the sample size, both subgroups showed that a lower PNI was associated with poorer OS according to the pooled HR (*p* < 0.001), and no significant heterogeneity observed. This result further supported the predictive impact of the PNI in patients with HNC. Additionally, in the subgroup analyses, we found that the associations of PNI with OS were not greatly affected by the PNI cutoff value or analysis method, implicating that PNI is a recognized serum marker for predicting the prognosis of HNC.

It’s worthy to note that a significant publication bias was recognized in the present study. Funnel plots showed that studies with higher standard errors tended to report higher HRs than studies with lower standard errors, which may indicate a small study effect. We performed the trim and fill method and found that the pooled HR stayed constant, which further supported the predicted value of the PNI. Furthermore, subgroup analysis indicated that the predictive performance and sensitivity of the PNI did not change according to the sample size, further demonstrating the stability and robustness of our analysis.

This meta-analysis has some limitations and should be considered. First, the cutoff values of PNI are vary through include studies. The cutoff value recognized by one cohort study could not reproduce in other independent cohorts. This makes this biomarker more difficult to apply in clinical practice.

Till now, the relationship between host immunity and tumour cells has not been fully understood, and inflammation definitely has an important role and has been elucidated as a hallmark of cancer. Second, most of the included studies came from Asia (Japan, China and Turkey), and only one Western dataset was included (from Spain); therefore, it may not represent real-world data. Third, most of the studies were observational and retrospective. Potential selection bias may exist. Finally, there was significant publication bias in our meta-analysis, which may be caused by the publication of positive studies or small study effects. However, the result did not change after adjusting for bias; hence, our data still provide sufficient capacity to evaluate the relationship between the PNI and the survival of HNC. Nevertheless, prospective randomized controlled studies with adequate sample sizes are still needed to validate our findings.

## Materials and methods

### Data source and search strategy

This systematic review and meta-analysis was performed based on the Preferred Reporting Items for Systematic Reviews and Meta-analysis (PRISMA) criteria^41^. The study waived ethical approval because it does not include individual patient information. A structured online search using the U.S. National Library of Medicine (PubMed), the Cochrane Central Register of Controlled Trials (CENTRAL), and the Excerpta Medica database (Embase) for all available studies from 1966 through June 2020. Keywords used for the search included “(Prognostic nutritional index) AND (nasopharyngeal OR oropharyngeal OR laryngeal OR hypopharyngeal OR oral OR head OR neck) AND (cancer OR squamous cell carcinoma OR tumour OR neoplasm)”. The clinical trials database (http://clinicaltrial.gov) was also searched for ongoing clinical trials. We also manually screened the titles, abstracts, full texts, and reference lists of the extracted articles to identify more potential eligible studies.

### Inclusion and exclusion criteria

Published studies that met the following inclusion criteria were included: (1) the study determined the association between pretreatment PNI and survival outcome, including overall survival (OS), disease-free survival (DFS), disease-specific survival (DSS), progression-free survival (PFS) or distant metastasis-free survival (DMFS) in HNC; (2) those studies included patients who did not receive any oncologic therapy, such as neoadjuvant therapy or surgery, before enrolment; and (3) those articles provided adequate details for calculation or data extraction of the individual hazard ratio (HR) or odds ratio (OR) and corresponding 95% confidence intervals (CIs). Two authors (C.W.L. and K.Y.C.) independently included the studies identified from the searches if they met the inclusion criteria. All the references from the included studies were also reviewed to determine any additional related studies. A third author (Y.T.T.) adjudged when the two authors disagreed. The exclusion criteria were as follows: (1) those trials did not include survival outcomes or (2) the studies included patients with metastatic disease or (3) the article was a letter, epidemiological study, case report, review article, meta-analysis, conference abstract, or duplicate publication.

### Data extraction and quality assessment

Two authors (C.W.L. and K.Y.C.) independently extracted data from the included trials using the same data collection form. The data included study details (country of study, first author, year of publication and sample size), pathological characteristics (TNM staging) and clinical features (survival outcome, PNI cut-off values, type of treatment applied, and duration of follow-up period). A third author (Y.T.T.) arbitrated when the 2 authors disagreed.

### Quality assessment

The quality assessment of the included trials was determined by the same two investigators independently based on the Newcastle–Ottawa quality assessment scale (NOS)^42^. A third author (Y.T.T.) arbitrated when the 2 authors disagreed. We considered NOS values greater than 6 to be high-quality studies.

### Statistical analysis

The primary outcome was OS in patients with HNC. The secondary outcomes included DSS, DFS, PFS and DMFS. We presented risk differences with pooled HRs and corresponding 95% CIs to determine the correlation between PNI and survival outcomes of HNC. The heterogeneity among the eligible trials was calculated by the Cochran *Q*-test and *I*^2^ statistic^44^. *I*^2^ values of 0% to 24.9%, 25% to 49.9%, 50% to 74.9%, and 75% to 100% were considered no, low, moderate, and high heterogeneity, respectively^43,44^.

A random-effects model was used for all outcomes because clinical heterogeneity across the included trials^45^ was highly likely. To assess publication bias, we generated a funnel plot with Begg’s and Egger’s tests^46,47^, and *p* > 0.05 was considered to indicate no publication bias. Statistical significance was defined as *p* < 0.05. All statistical analyses were conducted with R, version 3.6.2, using the “metafor”^48^ and “meta”^49^ packages for meta-analysis.

## Conclusions

In conclusion, our study suggests that a low PNI is correlated with poor OS, PFS, DFS, DSS and DMFS in patients with HNC. Apart from the above limitation, our data support that a low PNI can be a valuable prognostic indicator in patients with HNC. Further research including large prospective studies is required to confirm the association between pretreatment PNI and the survival outcome of patients with HNC.

## Supplementary Information

Supplementary Information.
